# Autologous transplantation of conjunctival epithelial cells cultured on amniotic membrane in a rabbit model

**Published:** 2007-07-13

**Authors:** Kyoko Ono, Seiichi Yokoo, Tatsuya Mimura, Tomohiko Usui, Kazunori Miyata, Makoto Araie, Satoru Yamagami, Shiro Amano

**Affiliations:** 1 Department of Ophthalmology, University of Tokyo Graduate School of Medicine, Tokyo, Japan; 2 Department of Corneal Tissue Regeneration, University of Tokyo Graduate School of Medicine, Tokyo, Japan; 3 Miyata Eye Hospital, Miyakonojo, Miyazaki, Japan

## Abstract

**Purpose:**

To evaluate the feasibility of autologous transplantation in a rabbit model of conjunctival epithelial cells cultured on amniotic membrane for ocular surface reconstruction.

**Methods:**

Limbal stem cell deficiency was induced in the right eyes of 30 rabbits. This was done by performing a lamellar keratectomy of the entire cornea and a complete removal of the limbus and conjunctiva, extending 5 mm outside the limbus. Autologous conjunctival specimens were obtained from the left eyes of ten of those rabbits and cultured for four weeks on denuded amniotic membrane. Cultured epithelium was examined by transmission electron microscopy. Four weeks after lamellar keratectomy, conjunctivalized corneal surfaces were excised and autologous cultured conjunctival epithelial sheets transplanted (Conj-AM group, n=10). The controls were rabbits that underwent corneal surface removal but not transplantation (No Transplantation group, n=10) and those that underwent corneal surface removal but received only amniotic membrane (AM Alone group, n=10). A neovascularization and corneal opacity scoring system was used to evaluate each eye in the two months after surgery.

**Results:**

Cultured conjunctival epithelium formed three to four layers on denuded amniotic membrane. Averaged scores of corneal neovascularization and corneal opacity two months after transplantation were significantly low in the Conj-AM group as compared with those in the AM and no transplantation groups.

**Conclusions:**

Transplantation of autologous conjunctival epithelial cells cultured on amniotic membrane should prove an effective strategy for treating total limbal stem cell deficiency.

## Introduction

Total conjunctivalization of the corneal surface seriously impairs vision in eyes with total limbal stem cell deficiency (LSCD), such as Stevens-Johnson syndrome, chemical burns, cicatricial pemphigoid, and aniridic keratopathy. Allolimbal transplantation [1-4] and transplantation of cultured limbal [5-8] or oral [9-11] epithelium have been proposed as LSCD therapies, but each has intrinsic disadvantages. All methods that use allotissues or cells require intense systemic immunosuppressive therapy after surgery. Moreover, the transplantation of cultured autologous limbal epithelium requires a healthy limbus in the contralateral eye whereas transplantation of cultured autologous oral epithelium induces peripheral corneal neovascularization [9-11]. The long-term outcomes of these therapies, however, have yet to be clarified. We report the feasibility of transplantation of auto conjunctival epithelium cultured on amniotic membrane.

## Methods

### Rabbit model of total limbal stem cell deficiency

All procedures were performed according to the ARVO Statement for the Use of Animals in Ophthalmic and Vision Research. The right eyes of 30 New Zealand white rabbits (2-2.5 kg; Saitama Experimental Animal, Saitama, Japan) were anesthetized by intramuscular injection of 20 mg/ml xylazine hydrochloride (Bayer, Bayer, Leverkusen, Germany) and 50 mg/ml ketamine hydrochloride (Sankyo, Tokyo, Japan), Next, total LSCD was created by performing lamellar keratectomy of the entire cornea and total removal of the limbus and conjunctiva, extending 5 mm beyond the limbus.

### Preparation of human amniotic membrane

The preparation of human amniotic membrane protocol was approved by the institutional review board of Miyata Eye Hospital and Noda Obstetrics and Gynecology Clinic (Miyakonojo, Miyazaki), and performed in accordance with the Helsinki Declaration of 1975 and its 1983 revision. Informed consent was obtained from each patient prior to harvesting human amniotic membrane at the time of Caesarean section (two patients). Under sterile conditions, the membrane was washed with sterile phosphate-buffered saline (PBS) containing 0.1 mg/ml streptomycin (Sigma-Aldrich, St. Louis, MO) and 0.25 μg/ml amphotericin B (Sigma-Aldrich). It was then cut into approximately 3 cm x 3 cm pieces and stored at -80 °C in a cell banker (Wako Pure Chemical Industries, Osaka, Japan). Before use, it was thawed, washed three times with sterile PBS, after which amniotic epithelial cells were removed by incubation for 2 h with 0.02% EDTA (Wako Pure Chemical Industries) at 37 °C, then with gentle abrasion with a cell scraper (Corning Inc., Corning, NY).

### Conjunctival epithelial cell culture on amniotic membrane

The 3T3 fibroblasts were incubated with 4 μg/ml mitomycin C at 37 °C for 2 h, rinsed with PBS, tripsinized, then placed in 6-well plastic dishes (Corning) at a density of 2x10^4^ cells/cm^2^. After being rinsed in PBS, the denuded amniotic membrane was spread, epithelial basement side up, on culture plate inserts (Corning), then the inserts were placed in the dishes containing the treated 3T3 fibroblasts. Conjunctival tissues, approximately 2 mm x 4 mm, obtained from the conjunctival fornix and bulbar conjunctiva of the normal left eyes of 10 rabbits, were washed in 10% iodine then rinsed with PBS. The conjunctival epithelium was removed from the stroma by mechanical scraping and was placed on denuded amniotic membranes and cultured in medium consisting of DMEM and Ham's F12 (in a 1:1 ratio; Sigma-Aldrich) supplemented with 10% fetal bovine serum, 20 ng/ml epidermal growth factor (Sigma-Aldrich), 5 μg/ml human recombinant insulin (Sigma-Aldrich), 0.1 μg/ml cholera toxin (Sigma-Aldrich), penicillin-streptomycin, and amphotericin B (Sigma-Aldrich). The medium was changed every three days and culture maintained for one month before use.

### Transplantation of autologous conjunctival epithelium cultured on amniotic membrane

One month after LSCD induction, the entire corneal surface was covered with conjunctival tissue, and there was neovascularization in all 30 eyes. The right eyes of 20 rabbits underwent 360° peritomy followed by removal of the fibrovascular membrane on the cornea. Autologous conjunctival epithelium cultured on amniotic membrane was transplanted to 10 rabbits (Conj-AM group). In the other 10 rabbits, only denuded amniotic membrane was sutured on the denuded cornea and limbus (AM Alone group). As the other experimental control, 10 eyes with conjunctivalized cornea underwent removal of the fibrovascular membrane on the cornea but no transplantation (No Transplantation group). After surgery, topical 0.5% levofloxacin (Santen Pharmaceutical, Osaka, Japan) and 0.1% phosphate betamethasone (Shionogi Pharmaceutical, Osaka, Japan) were applied three times daily for one month.

At one week, one month, and two months after surgery, the corneal surface was viewed under a surgical microscope. Consequent scoring was done by the same examiner in masked fashion. Scoring of the degree of corneal neovascularization depended on the maximum reach of the invasion of corneal neovascularization: grade 0, no neovascularization; grade 1, maximum reach less than one-third the distance between the limbus and corneal center; grade 2, maximum reach between one- and two-thirds the distance between the limbus and corneal center; grade 3, maximum reach more than two-thirds the distance between the limbus and corneal center; grade 4, maximum invasion reaching the corneal center. The degree of corneal clarity was scored as follows: grade 0, totally clear; grade 0.5, trace or faint corneal haze; grade 1, mild haze of minimal density; grade 2, moderately dense opacity partially obscuring the pupil; grade 3, severely dense opacity completely obscuring the pupil.

### Histological examination

Conjunctival epithelial cells that had been cultured on amniotic membranes for one month were examined histologically. Samples were fixed in buffered 8% paraformaldehyde, embedded in paraffin, cut to 4 μm thickness, and placed on MAS-coated microscope slides (Matsunami Glass Inc, Tokyo, Japan). They were then stained with hematoxylin and eosin and examined under a light microscope.

### Electron microscopy

Conjunctival epithelia cultured on amniotic membranes were immersed for 10 min in fixative consisting of 2.5% glutaraldehyde in 0.1 M PBS at pH 7.4, segmented in the same fixative, then reimmersed in fixative for 12 h, after which they were osmicated in 1% OsO4 in the buffer for 1 h, dehydrated in ethanol, and embedded in Epon 812. Sections were cut 1 μm thick with glass knives and stained with toluidine blue. Ultrathin sections were cut and stained with lead citrate and uranyl acetate and examined in a transmission electron microscope (Model 100C, Nihon Denshi, Tokyo, Japan).

## Results

### Culture of conjunctival epithelial cells

**Figure 1:**
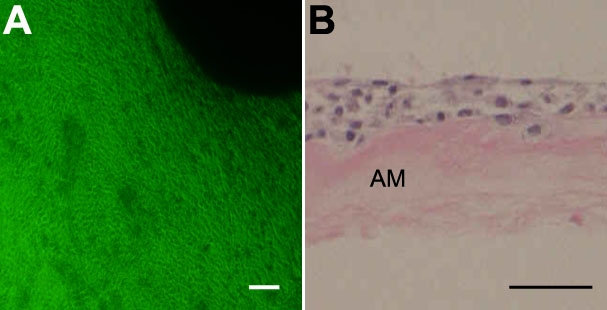
Rabbit conjunctival epithelial cells cultured on amniotic membrane. **A**: Epithelial cells became confluent within 14 days. **B**: Epithelial cells formed three to five layers without goblet cells on amniotic membrane after four weeks of culture. H&E and PAS staining. AM=amniotic membrane. The scale bar in **A** equals 100 μm and the scale bar in **B** equals 50 μm.

**Figure 2:**
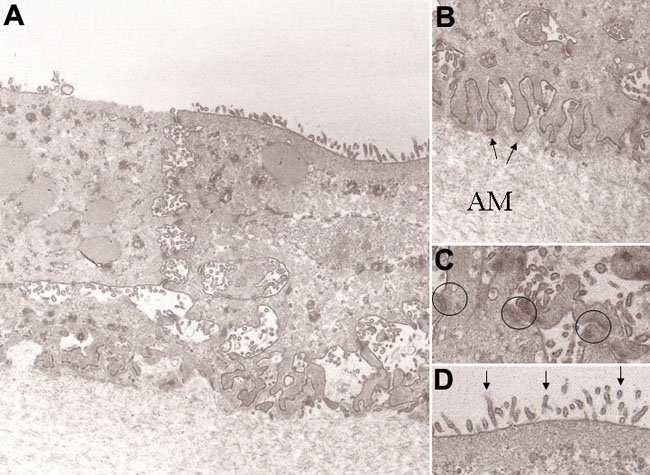
Transmission electron micrographs of rabbit conjunctival epithelial cells grown on amniotic membrane. **A**: After 4 weeks of culture, epithelial cells formed three to five layers without goblet cells on amniotic membrane. Original magnification: X3,000. **B**: Attachment of epithelial cells to the amniotic membrane basement membrane by hemidesmosomes (arrows). AM=amniotic membrane. Original magnification: X40,000. **C**: Adjacent epithelial cells were joined by numerous desmosomes (circles). Original magnification: X40,000. **D**: Many microvilli (arrows) of superficial cells were present on the cultured conjunctival epithelium. Original magnification: X40,000.

Epithelial cells from conjunctival specimens began to propagate on human amniotic membrane within three days and became confluent within two weeks (Figure 1A). They were well stratified in three to four layers without goblet cells four weeks after propagation (Figure 1B, Figure 2A). Transmission electron microscopy of the conjunctival epithelial culture sheet showed that the basal epithelial cells adhered to the amniotic membrane substrate with hemidesmosomes (Figure 2B), and adjacent cells were well attached to each other by numerous desmosomes (Figure 2C). Microvilli were abundant on the surfaces of the superficial cells (Figure 2D). Such cell-cell and cell-substrate adhesion structures are crucial for maintaining cell sheet integrity.

### Outcome of autologous conjunctival epithelial sheet transplantation

**Figure 3:**
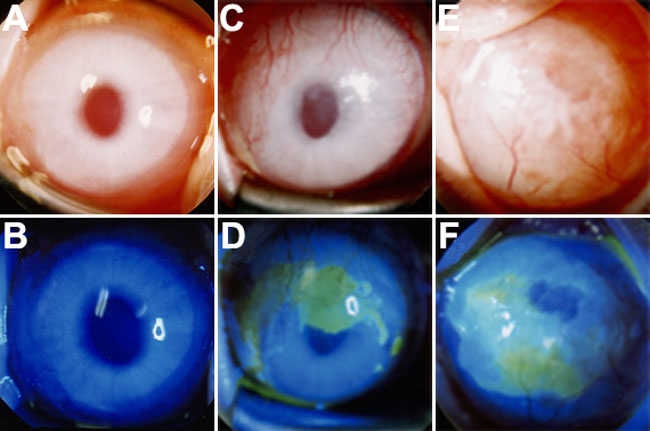
Representative cases from the three groups at two months after surgery. In most of the cases in the Conj-AM group, the cornea was clear with minimal neovascularization and had no epithelial defects (**A** and **B**). In the AM Alone group, moderate neovascularization and opacity was present in the midperipheral to peripheral cornea and epithelial defects in the central area (**C** and **D**). In the No Transplantation group, advanced neovascularization and opacity were present throughout the cornea, and epithelial defects were in the central area (**E** and **F**).

**Figure 4:**
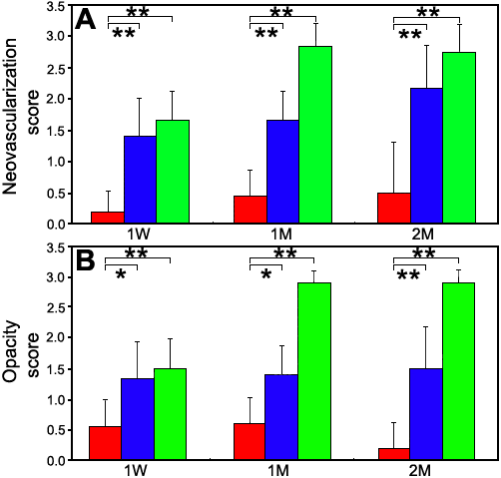
Corneal neovascularization and opacity scores one week, one month, and two months after surgery. Averaged corneal neovascularization (**A**) and opacity (**B**) scores in the Conj-AM group (red columns) are significantly low at all times as compared with those in the AM Alone (blue) and No Transplantation (green) groups. ANOVA and Fisher's PLSD. The asterisk indicates a p<0.05 and the double asterisk indicates a p<0.0001.

Representative cases from the three groups at two months after surgery are shown in Figure 3. In most of the cases in the Conj-AM group, the cornea was clear with minimal neovascularization and without epithelial defects. In contrast, the AM Alone group showed moderate neovascularization and opacity in the midperipheral to peripheral cornea and epithelial defects in the central area. In the No Transplantation group, advanced neovascularization and opacity were present throughout the cornea, and there were epithelial defects in the central area. Corneal neovascularization and clarity scores are shown in Figure 4. At all evaluation times, scores for corneal neovascularization and opacity were significantly lower in the Conj-AM group than those of the AM Alone and No Transplantation groups.

## Discussion

In our study, transplantation of autologous conjunctival epithelium cultured on amniotic membrane effectively restored a clear corneal surface with minimum neovascularization, whereas corneas in the control groups with transplanted amniotic membrane only or no transplant had considerable neovascularization and opacity with epithelial defects. Transplantation of autologous conjunctival epithelium cultured on human amniotic membrane therefore may provide a method of choice for treating eyes with LSCD.

Such transplantation may prove especially useful for patients with bilateral LSCD, for whom transplantation of autologous cultivated limbal epithelium is impossible. The feasibility of autologous oral mucosal epithelial transplantation has been shown [9-11], but the long-term outcome has yet to be clarified. Also, conjunctival epithelium gene expression is believed to be closer to that of the corneal epithelium than that of the oral mucosal epithelium, indicative of the conjunctival epithelium being superior for restoring the corneal surface. Clinical studies with long-term follow-ups can help determine which of these two methods, utilization of autologous conjunctival or oral epithelium, is superior.

Recently, Tanioka et al. [12] reported that human conjunctival epithelial cells cultured on human amniotic membrane and transplanted onto denuded rabbit corneas were effective in maintaining corneal transparency for two weeks. While their study demonstrated the potential of cultured human conjunctival epithelial cells as an alternative tissue source for replacement of the corneal epithelium, their model was a xenotransplantation with a relatively short observation period. Our model is an autotransplantation with a longer observation period that is more clinically relevant and confirms the potential of cultured conjunctival epithelium as a cell source for ocular surface reconstruction.

In this study, conjunctival epithelium was harvested from healthy conjunctiva of young rabbits. Patients with LSCD, however, often have diseased conjunctiva, and those with Stevens-Johnson syndrome or ciciatric pemphigoid frequently have severe symblepharon, making harvest of conjunctival tissue difficult. Moreover, LSCD patients often are elderly, for whom the culture of conjunctival epithelial cells is more difficult than for younger persons. The indication for transplantation of conjunctival epithelium cultured on amniotic membrane therefore may be limited to bilateral mild to moderate LSCD in relatively young patients who have aniridia keratopathy, Stevens-Johnson syndrome, or chemical burns.

If transplantation of cultured conjunctival epithelium is to be clinically feasible, two points must be clarified. First, longer-term results of such transplantation need to be examined. Gradual corneal neovascularization has been reported after transplantation of oral mucosal epithelium [9-11]. Because the conjunctival epithelium is more pro-angiogenesis than the corneal epithelium [13], similar corneal neovascularization is expected after transplantation of cultured conjunctival epithelium. Second, conjunctival epithelium should be harvested from sites that include tissue stem cells. We harvested conjunctival epithelium from the conjunctival fornix and bulbar conjunctiva. The conjunctival fornix [14-17], bulbar conjunctiva [18], and mucocutaneous junction conjunctiva [19,20] have been proposed as having tissue stem cells of the conjunctival epithelium, but the site of these tissue stem cells is disputed; therefore, it must be determined where conjunctival tissue will be harvested.

In conclusion, transplantation of autologous conjunctival epithelial cells cultured on amniotic membrane was effective for at least two months for restoration of transparent corneas in rabbit eyes with LSCD. Longer-term observations using this rabbit model are required before this method can be adopted for clinical treatment.
